# Mummified Cats

**Published:** 1890-04

**Authors:** 


### MUMMIFIED CATS.

A writer in the London *Daily Telegraph* gives an account of a consignment of nineteen and a half tons of mummified cats from Beni Hassan, Central Egypt, which has just reached Liverpool, being perhaps the most curious cargo that has ever been carried across the seas. There are in the parcel the remains of about 180,000 grimalkins, and they are in process of examination at the city Museum. The public will be curious to learn the history of this strange find, and as so far told it is as romantic as a page of Rider Haggard. The "harmless, necessary cat," so by Shakespeare styled in "The Merchant of Venice," was as familiar in ancient Egypt as in modern Europe, though in the times long past the tabby played a more honored part. The feline mausoleum, as we hear, was discovered by an Egyptian peasant engaged in the pursuit of husbandry, who one day fell unwarily into a pit that he had not digged. It was found that the excavation was in reality a large subterranean cave—a sepulchre of cats, laid out in rows, every one of which had been separately embalmed, like the bodies in the fabled caverns of Kor. The date of this extraordinary interment is fixed by Egyptologists at two thousand years B. C. It is a well known fact that in ancient Egypt the cat was held in reverence, and there has been a tradition that a cemetery devoted specially to the interment of the species existed on the east bank of the Nile. The locality has now been fixed, and it is about one hundred miles distant from Cairo. From this graveyard enormous quantities of feline remains have been exhumed. Part of the store was sold to local farmers for purposes of fertilization, and, as we are informed, other lots found their way to an Alexandrian merchant, thence by the steamers Pharos and Thebes to Liverpool, where the stock was knocked down at £2 13s 9d per ton to a manure merchant, the broker using a cat's head for a hammer. It is marvelous that after the lapse of so many centuries the superstitions of an ancient race should be made the manure for the bread of the latter days.

